# Supplementary material for: A Critical View of the Application of the APEX Software (Aqueous Photochemistry of Environmentally-Occurring Xenobiotics) to Predict Photoreaction Kinetics in Surface Freshwaters
Source: Molecules. 2019 Dec 18;25(1):9. doi: 10.3390/molecules25010009 (PMC7017383; doi:10.3390/molecules25010009)
Supplement: Supplementary file 1 [file molecules-25-00009-s001.zip › 3.2.4_gcc-4.4.0.zip!/3.2.4_gcc-4.4.0/3.2.4_gcc-4.4.0/doc/octave/html/interpreter/Simple-Output.html]

Simple Output - Untitled


Next: Line-Oriented Input,
Previous: Opening and Closing Files,
Up: C-Style I/O Functions


---

#### 14.2.2 Simple Output

Once a file has been opened for writing a string can be written to the
file using the `fputs` function. The following example shows
how to write the string Free Software is needed for Free Science
to the file free.txt.

```
     filename = "free.txt";
     fid = fopen (filename, "w");
     fputs (fid, "Free Software is needed for Free Science");
     fclose (fid);
```

— Built-in Function: **fputs** (fid, string)  
> Write a string to a file with no formatting.
>
> Return a non-negative number on success and EOF on error.
>
>
>
> **See also:** scanf, sscanf, fread, fprintf, fgets, fscanf.

A function much similar to `fputs` is available for writing data
to the screen. The `puts` function works just like `fputs`
except it doesn't take a file pointer as its input.

— Built-in Function: **puts** (string)  
> Write a string to the standard output with no formatting.
>
> Return a non-negative number on success and EOF on error.
